# Fumarate hydratase-deficient renal cell carcinoma in extended remission with bevacizumab and erlotinib

**DOI:** 10.3332/ecancer.2022.1404

**Published:** 2022-05-30

**Authors:** Shubham Tomar, Lakhan Kashyap, Akhil Kapoor

**Affiliations:** Department of Medical Oncology, Mahamana Pandit Madan Mohan Malviya Cancer Centre and Homi Bhabha Cancer Hospital, Tata Memorial Centre, Varanasi, Uttar Pradesh 221005, India

**Keywords:** hereditary leiomyoma and renal cell carcinoma, FH gene mutation, bevacizumab and erlotinib therapy

## Abstract

Hereditary leiomyomatosis and renal cell carcinoma (HLRCC) is an autosomal dominant syndrome associated with fumarate hydratase (FH) gene mutation leading to defective DNA double-strand break repair mechanism. Although these tumours have an aggressive presentation, they respond well to targeted therapy with fewer adverse effects. Here we present a case of a 42-year-old female having isolated renal cell carcinoma, papillary type 2, carrying a mutation in the FH gene without cutaneous and uterine involvement. Her tumour responded well to erlotinib and bevacizumab combination and she was on treatment for 23 months. This report adds to the current literature and can help to define treatment protocols for HLRCC.

## Introduction

Hereditary leiomyomatosis and renal cell carcinoma (HLRCC) is an autosomal dominant deoxyribonucleic acid (DNA) repair-deficiency syndrome characterised by cutaneous and uterine leiomyomas and increased risk of developing renal cell carcinoma [1]. The fumarate hydratase (FH) gene is a tumour suppressor gene present on chromosome 1 that codes for the fumarase enzyme of the tricarboxylic acid (TCA) cycle and is also involved in repairing DNA double-strand breaks [2]. Renal cell carcinoma in patients with FH-mutated HLRCC is known to be aggressive with early distant metastasis. Early surveillance is recommended for suspected individuals, and a multisystem management approach is required for HLRCC cases. We describe a case of a 42-year-old female having primary renal cell carcinoma and multiple distant metastases without any cutaneous or uterine lesion. This case underscores the present recommendation for genetic testing and tumour surveillance in isolated renal cell carcinomas and adds to the current treatment strategies.

## Case report

A 42-year-old female of Asian descent presented to Tata Memorial Hospital with complaints of haematuria and burning micturition for the past 2 months. An abdominal ultrasound revealed a sizable hypoechoic mass in the mid-lower pole of the left kidney, involving renal pelvis and calculous cholecystitis. Further evaluation with 18F-fluorodeoxyglucose positron emission tomography/computed tomography (CT) confirmed a metabolically active 9.7 x 6.7 cm mass lesion, involving the left kidney with exophytic extension inferiorly and into the renal pelvis. The tumour extended into the left renal vein but did not involve the inferior caval vein. The patient also had multiple nodal involvements and distant metastases to the bilateral lung and segments seven and eight of the liver. Biopsy from the renal mass revealed vague papillary architecture with tumour cells having abundant eosinophilic cytoplasm and large hyperchromatic nuclei suggestive of renal cell carcinoma, papillary type 2. On immunohistochemistry, the tumour was positive for AMACR, Vimentin and PAX8, and negative for CK7. Complete blood count, liver function test, renal function test, serum electrolytes and calcium were within the standard limits. Because of extensive metastasis, surgical intervention was not performed, and the patient was planned for systemic therapy.

Germline genetic testing was carried out given the histopathology and patient age, revealing a FH gene mutation in exon 6 of chromosome 1, which predisposes to hereditary renal cancer. The patient had no cutaneous nodules, and abdominopelvic sonography showed no leiomyoma in the uterus. The patient was treated with tab. erlotinib 100 mg once daily and inj. bevacizumab 500 mg at 10 mg/kg since November 2019 and received 46 cycles biweekly during a 23 months follow-up period. While on treatment, she developed recurrent episodes of erlotinib-induced grade 3 acneiform rash, which were managed by withholding erlotinib and starting doxycycline (100 mg twice a day as and when required). The patient also developed an episode of acute calculous cholecystitis during treatment and underwent lap cholecystectomy in March 2020. The treatment was interrupted for 8 weeks during this time.

**Figure 1.**
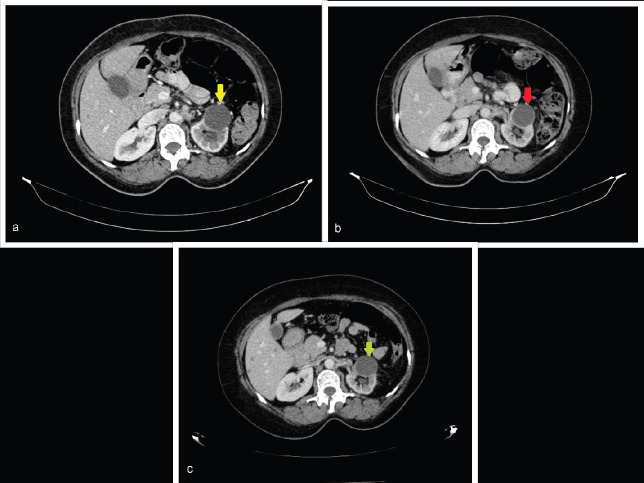
Representative axial cut images of the abdominopelvic contrast-enhanced computed tomography (CECT) scan. (a): CECT performed in July 2020 showing a mild interval decrease in left kidney hypo-enhancing lesion from 41 × 38 mm to 33 × 29 mm (yellow arrow) and similarly a decrease in size of the retroperitoneal node from 20 × 16 mm to 19 × 10 mm. (b): CECT performed in December 2020 showing a decrease in size in the left kidney hypo-enhancing lesion from 33 × 29 mm to 28 × 26 mm (red arrow). (c): CECT performed in September 2021 showing significant reduction in size of renal mass from 41 × 38 mm to 28 × 24 mm (green arrow) and retroperitoneal nodes from 20 × 16 mm to 16 × 9 mm.

Response CT scan in July 2020 (Figure 1a) showed stable disease as per Response Evaluation Criteria in Solid Tumours 1.1. A follow-up CT scan in December 2020 (Figure 1b) showed further regression of the tumour. She is alive and her disease was stable until the last follow-up in October 2021, and she planned for maintenance therapy with oral erlotinib 100 mg once daily and injectable bevacizumab 500 mg at 10 mg/kg biweekly. Her latest CT scan in September 2021 continued to show regression in tumour burden (Figure 1c).

## Discussion

**Figure 2.**
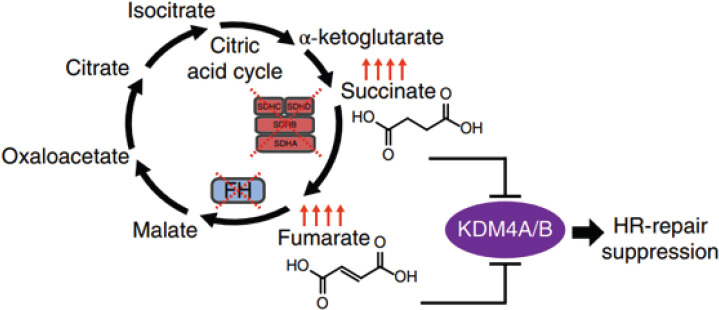
Model of succinate and fumarate-induced HR DNA repair pathway suppression [2].

HLRCC is an autosomal dominant syndrome recently recognised in the WHO 2016 classification and is characterised by germline mutation in FH gene [3]. Mutation in the FH gene leads to the build-up of fumarate, which further activates transcription factors responsible for inappropriate stimulation of oncogenic hypoxia [4]. Fumarate and succinate are metabolites of TCA cycles that inhibit α-ketoglutarate -dependent dioxygenases that are responsible for histone demethylation or promote DNA demethylation. This can compromise the homologous recombination (HR) DNA double-strand repair pathway, which is required for the maintenance of genomic integrity (Figure 2) [2, 5]. FH-deficient RCC is a variant of HLRCC with no evidence of FH germline mutation; however, it is characterised by the absence of the FH gene among tumour cells [3].

Our patient developed symptoms at the age of 42 years with only renal involvement. Not all patients of HLRCC develop renal cell carcinoma. The mean age at detection is around 37 years, and the patient presents with haematuria, low back pain and/or a palpable mass [6]. As described by Launonen *et al* [7], ‘RCC related with HLRCC are characterised histologically by the presence of cells with abundant amphophilic cytoplasm and large nuclei with large inclusion like eosinophilic nucleoli’. A morphological variant of FH-deficient RCC with low-grade nuclei and eosinophilic cytoplasm has a better outcome [3]. Negative expression of FH and positive expression of 2-succinyl-cysteine are sensitive and specific indicators of fumarase hydratase-deficient renal cell carcinoma [8].

Our patient only met one minor criterion described by the study conducted by Schmidt *et al* [9]. This study proposed the diagnostic criteria for HLRCC which included one major and three minor criteria. According to Schmidt *et al* (9), ‘Major criteria point towards a high possibility of HLRCC and includes multiple cutaneous leiomyoma (CLM), out of which one is histologically confirmed leiomyoma. The minor criteria raise suspicion for HLRCC and include (a) solitary CLM and family history, (b) early-onset type 2 papillary tumour of the kidney and (c) multiple early-onset symptomatic uterine fibroids (<40-year-old)’.

Early surveillance is recommended for individuals suspected of HLRCC. A dermatological examination every 2 years to look for CLM transformation is recommended. After the age of 15 years, annual gynaecological examination to look for ULM and counselling regarding the risk of hysterectomy and family planning by 18 years is recommended. From age 10 years, annual contrast-enhanced magnetic resonance imaging of the kidney to look for early-stage RCC is recommended [10].

A multidisciplinary approach to management is required for HLRCC cases. Asymptomatic CLM requires no treatment, while cryoablation/surgical excision can be performed for solitary lesions. Medications like calcium channel blockers, nitroglycerine, alpha blockers and anti-epileptic can reduce pain associated with leiomyomas [11]. Medical therapy, including hormone therapy and gonadotropin-releasing hormone agonist, can be used for initial management of uterine leiomyomas. Because of rapid growth and multiplicity, most women require surgical interventions [12]. HLRCC-associated RCC is an aggressive tumour and requires early surgical excision. Radical nephrectomy or partial nephrectomy with wide margins should be considered for renal masses [13].

Other therapies such as poly(ADP-ribose)polymerase inhibitors, hypoxia-inducible factor 1-alpha targeting agents and anti-angiogenic agents targeting aerobic glycolysis are under study. The AVATAR trial, a phase II prospective study by Srinivasan *et al* [14], showed that the combination of bevacizumab and erlotinib is used to reduce glucose delivery to tumour cells and is well tolerated in patients with FH-deficient tumours with an overall response rate (ORR) of 51% and median progression free survival (PFS) of 14.2 months among all patients. Another retrospective study by Carril-Ajuria *et al* [15] suggested that anti-angiogenic agents were superior to immune checkpoint inhibitors and mammalian target of rapamycin (mTOR) inhibitors in FH-def RCC. Various treatment-related adverse events associated with this combination are acneiform rash, diarrhoea and skin dryness. Rarely, gastrointestinal haemorrhage can be related to bevacizumab [14]. In a study by Gleeson *et al* [16], the vascular endothelial growth factor (VEGF)/mTOR combination led to a higher ORR of 44% than that observed with anti-angiogenic monotherapy (ORR of 20%). Presently, there is no Food and Drug Administration (FDA)-approved therapy for HLRCC and the NCCN guidelines recommend VEGF inhibitor/tyrosine kinase inhibitor (TKI) for non-clear cell histology of renal cell carcinoma. However, non-clear cell RCC behaves biologically different from clear cell variant, and non-clear cell RCC may not respond favourably to these therapies. In the absence of established guidelines and favourable data for a bevacizumab and erlotinib combination from phase II studies, we selected this regimen for our patient.

## Conclusion

HLRCC is a rare autosomal dominant disease with an aggressive presentation and early distant metastasis. This case report highlights the less common presentation of FH-mutated RCC and the importance of TKI/anti-VEGF combination in its treatment. The combination of erlotinib and bevacizumab is effective with moderate side effects and could be recommended as the standard of care.

## Conflicts of interest

None declared.

## Funding

Nil.
